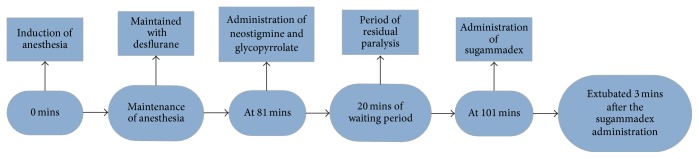# Management of Residual Neuromuscular Blockade Recovery: Age-Old Problem with a New Solution

**DOI:** 10.1155/2017/8197035

**Published:** 2017-03-14

**Authors:** Michael S. Green, Archana Gundigi Venkatesh, Ranjani Venkataramani

**Affiliations:** Department of Anesthesiology and Perioperative Medicine, Drexel University College of Medicine, 245 N. 15th Street, Suite 7502, MS 310, Philadelphia, PA 19102, USA

## Abstract

Neostigmine has been traditionally used as the agent of choice to reverse Neuromuscular Blockade (NMB) after muscle paralysis during general anesthesia. However, the use of neostigmine has not been without untoward events. Sugammadex is a novel drug that selectively binds to aminosteroid nondepolarizing muscle relaxants and reverses even a deep level of NMB. Controversy exists regarding the optimal dose of sugammadex that is effective in reversing the NMB after the incomplete reversal with neostigmine and glycopyrrolate. We discuss a case where sugammadex reduced the time of the recovery from NMB in a patient who had incomplete antagonisms following adequate treatment with neostigmine, aiding timely extubation without persistent residual NMB, and hence prevented the requirement of postoperative ventilation and the improvement in patient care. More randomized control studies are needed in order to conclude the appropriate dose of sugammadex in cases of incomplete reversal.

## 1. Introduction

Neostigmine has been traditionally used as the agent of choice to reverse Neuromuscular Blockade (NMB) after muscle paralysis during general anesthesia. However, the use of neostigmine has not been without untoward events, namely, in the form of postoperative residual paralysis. This residual Neuromuscular Blockade is due to incomplete antagonism of NMB medications. A train of four (TOF) ratios of 0.9 and above is indicative of adequate reversal from NMB. While quantitative assessment of neuromuscular recovery using TOF ratio is considered gold standard [1], most anesthesiologists do not have the ability to perform a quantitative assessment of neuromuscular function [2]. Other factors that influence recovery after NMB, although not exhaustive, are the duration of the paralytic agent, use of single or repeated doses [3], and depth of blockade at the time of administering anticholinesterase [4]. Sugammadex is a novel drug that selectively binds to aminosteroid nondepolarizing muscle relaxants and reverses even a deep level of NMB. There are many studies which proved the effectiveness of sugammadex in reversing the NMB immediately following administration of NMB. However, there is inadequate evidence of the effectives of sugammadex in cases of incomplete reversal with neostigmine and glycopyrrolate. Furthermore, controversy exists regarding the optimal dose of sugammadex that is effective in reversing the NMB after the incomplete reversal with neostigmine and glycopyrrolate. No standard dosing regimen exists yielding confusion on the management plan of residual curarization. Here, we discuss a case where sugammadex reduced the time of the recovery from NMB in a patient who had incomplete antagonisms following adequate treatment with neostigmine, aiding timely extubation without persistent residual NMB, and hence prevented the requirement of postoperative ventilation and the improvement in patient care. This case highlights sugammadex use in addition to neostigmine as an effective alternative in the management of patients with postoperative residual NMB.

## 2. Case Report

A 65-year-old female, 5′3′′ tall, weighing 52 kilograms with a non-small cell carcinoma of the left upper lobe presented for a staging mediastinoscopy and biopsy under general anesthesia. Her medical history was significant for hypertension, COPD, GERD, and hepatitis C. Preoperative laboratory evaluation values were all within normal limits.

Induction of anesthesia was performed with propofol 150 mg, fentanyl 75 mcg, and rocuronium 50 mg. Desflurane provided anesthesia maintenance. The procedure was uneventful with a total time of 81 minutes. Following confirmation of 3 twitches via TOF monitoring the patient received neostigmine 3 mg and glycopyrrolate 0.6 mg intravenously. Persistent fade assessed via visual estimation of the TOF response was still evident even 20 minutes after medication administration. An additional dose of neostigmine 1 mg and glycopyrrolate 0.2 mg was given intravenously. Following a waiting period of 15 minutes the patient still had residual neuromuscular weakness requiring mechanical ventilation support (Figure 1). The decision of mechanical ventilation postoperatively versus a sugammadex trial was considered.

Suspecting residual curarization, sugammadex at 2 mg/kg, total of 100 mg, was given intravenously. A dramatic improvement in clinical response in the form of improved muscle strength, head lift, and tidal volumes were noted. This was coupled with an absence of fade on eliciting a TOF response. Extubation was safely performed within the next 2 minutes and no further recurarization or residual NMB was seen in the PACU.

## 3. Discussion

Usage of muscle relaxants has brought several advantages in the field of anesthesiology such as optimizing surgical conditions, facilitating tracheal intubation, and improving mechanical ventilation. However, there are several disadvantages of using Neuromuscular Blocking Agents (NMBA) with the most critical one being inadequate recovery of neuromuscular function leading to postoperative pulmonary complications and upper airway muscle weakness. Hence, reversible agents such as acetylcholinesterase inhibitors and sugammadex are used in order to antagonize the effects of nondepolarizing muscle relaxants and to prevent the complications due to residual curarization [5].

Gaszynski et al. conducted a study which included morbidly obese patients undergoing general anesthesia for elective bariatric surgery. A total of 70 patients were allocated randomly into Group SUG where they received sugammadex for reversal of NMB and Group NEO where they received neostigmine for reversal. They found that mean time to 90% of TOF was 2.7 versus 9.6 minutes (*p* < 0.05) and TOF in the PACU was 109.2% versus 85.5% (*p* < 0.05) in Group SUG and Group NEO, respectively. This study proves that sugammadex is faster in reversing rocuronium-induced Neuromuscular Blockade compared to neostigmine [6].

Jones et al. conducted a study looking at the time taken for the recovery of NMB. This study included 37 patients in each study arm. One group received sugammadex of 4 mg/kg and the other group received neostigmine 70 *μ*g/kg along with glycopyrrolate of 14 *μ*g/kg for reversal of NMB. They found that sugammadex reversed the rocuronium-induced NMB within 2.9 mins as compared to 50.4 mins with neostigmine and glycopyrrolate. The authors concluded that sugammadex is 17-fold faster than the neostigmine and glycopyrrolate [7].

In our case, we used rocuronium of 1.0 mg/kg body weight for NMB at the time of induction. Rocuronium is a steroidal nondepolarizing muscle relaxant with duration of action ranging from 38 to 150 mins [8]. The surgical procedure was over in 81 mins and the patient was reversed with a standard dose of neostigmine and glycopyrrolate. We noticed a residual NMB even after the 20 minutes of neostigmine administration and showed significant fade on TOF stimulation along with inadequate tidal volume, poor respiratory efforts, and incoordination in hand movements. Given the clinical picture, we decided to administer sugammadex 100 mg instead of prolonged ventilation in order to prevent the complications associated with postoperative ventilation. After 2-3 minutes we noticed adequate tidal volume along with good respiratory efforts. There is very little data supporting the use of sugammadex following neostigmine administration. Neostigmine acts as a competitive antagonist at the neuromuscular junction by increasing the level of acetocholine available for binding to nicotinic receptors. Following the binding of the rocuronium by sugammadex there is no longer competition for the receptors thus leaving more available nicotinic and muscuranic receptors free for binding.

Cheong et al. conducted a study to compare the time to recovery of TOF ratio to 90% in four groups, Group S_2_ (2 mg/kg of sugammadex), Group S_1_ (1 mg/kg of sugammadex), Group SN (1 mg/kg of sugammadex and neostigmine 50 *μ*g/kg and glycopyrrolate 10 *μ*g/kg), and Group N (neostigmine 1 mg/kg + glycopyrrolate 10 *μ*g/kg). Study results showed time for the recovery of TOF to 90% was 182.6 ± 8, 371.1 ± 2, 204.3 ± 103.3, and 953.2 ± 3 seconds, respectively. This shows that 1 mg/kg of sugammadex along with neostigmine 50 *μ*g/kg and glycopyrrolate 10 *μ*g/kg reduced the time to 90% recovery of TOF significantly. Additionally, there was no clinically significant difference between the group which received sugammadex 2 mg/kg and the group which received 1 mg/kg of sugammadex along with neostigmine 50 *μ*g/kg and glycopyrrolate 10 *μ*g/kg. In our case, we used 2 mg/kg of sugammadex, which is a higher dose when compared to effective dose which was proved in this study, that is, 1 mg/kg [9]. However, there is inconclusive evidence supporting one dose versus another with regard to the dosage of combination therapy with sugammadex and neostigmine.

An additional study was conducted in order to see the incidence of residual NMB in patients who are reversed spontaneously, after neostigmine treatment and after sugammadex administration. Interestingly, TOF ratio of <0.9 was present in 4.3% of patients who were treated with sugammadex for reversal as compared to 13% in the spontaneous recovery group and 23.9% in patients who received neostigmine for reversal. This shows that no complete elimination of residual NMB occurs, even after the reversal with sugammadex. Hence, using neuromuscular monitoring plays a key role in diagnosing and treating the residual NMB [10]. Our case is a perfect example showing the importance of utilization of neuromuscular monitoring to diagnose the incomplete reversal from muscle relaxants as well as the need to monitor recovery from residual NMB. Intraoperative use of neuromuscular monitoring allows the anesthesiologist to use adequate muscle relaxants and antagonists during the operation. This is important because 10% of the anesthesiologists in the Europe and 20% of the anesthesiologist in the North America do not use neuromuscular monitoring [8].

In our case, there are many factors responsible for postoperative residual paralysis after rocuronium administration. First, there is an increase in the duration of action of rocuronium with age. A study conducted by Furuya et al. concluded that the duration of time taken for the appearance of posttetanic count (PTC) was twofold longer and variable in older as compared to younger individuals [11]. Secondly, there is a prolongation of rocuronium action in the female gender as compared to males [12]. Thirdly, there is a large variation in the duration of action of rocuronium itself [13]. Fourth, we used desflurane for maintenance of anesthesia which may prolong the action of rocuronium compared to other inhalational agents [14].

There was a similar case reported of a patient who developed postoperative residual NMB after single dose of rocuronium. The patient required ventilation for 125 mins following the end of surgery and was monitored until the complete recovery from NMB [13]. Here, we administered sugammadex 100 mg instead of postoperative ventilation after the diagnosis of incomplete reversal of NMB with neostigmine. The patient developed adequate muscle contraction with good respiratory efforts within 2-3 minutes and we were able to discharge the patient on the same day. This therapy proved to be cost-effective as there is early recovery from the residual NMB which saved the utilization of the anesthesiologists and money spent on ventilator management and prevented the complications of prolonged postoperative ventilation. Hence, this case very well illustrates the role of neuromuscular monitoring and the use of sugammadex in the case of residual paralysis. The best dosing scheme with the use of sugammadex following neostigmine administration still remains to be defined. Our choice of 2 mg/kg proved to be effective in this patient. The aforementioned study showed no difference between sugammadex 2 mg/kg and sugammadex 1 mg/kg along with neostigmine 50 *μ*g/kg and glycopyrrolate 10 *μ*g/kg. Further exploration of the use of sugammadex 2 mg/kg with neostigmine 50 *μ*g/kg for the reversal of neuromuscular blocking medications needs to be completed.

## 4. Conclusion

Sugammadex for reversing rocuronium-induced NMB has been used extensively in recent years. This novel cyclodextrin is used to reverse rocuronium-induced NMB in doses of up to 16 mg/kg. Recent studies from across the globe have been reporting success in reversing patients with variable depth of NMB by altering the dose of sugammadex.

We used a dose of 2 mg/kg in our patient, who demonstrated fade despite allowing for optimal duration of action of neostigmine. The dramatic recovery in neuromuscular function within 3 minutes of administration suggests that using this drug at lower doses such as 1-2 mg/kg in combination with neostigmine may perhaps prove to be a superior treatment modality. With its elevated price, sugammadex is somewhat cost prohibitive if not used scrupulously. However, if avoidance of postoperative ventilation is possible or postoperative pulmonary complications can be prevented the price of the medication will prove minimal. More studies are needed to pave the way for the administration of lower doses of sugammadex to supplement neostigmine in patients showing lighter levels of NMB or help serve as an alternative in patients showing fade or signs of inadequate NM recovery after reversal.

In our case, we demonstrated the advantages of using sugammadex in the case of residual NMB even after the reversal with neostigmine. In the future, more randomized control studies are need in order to conclude the appropriate dose of sugammadex in cases of incomplete reversal following neostigmine treatment.

## Figures and Tables

**Figure 1:**